# Point-of-care ultrasonography improves the diagnosis of splenomegaly in hospitalized patients

**DOI:** 10.1186/s13089-015-0030-8

**Published:** 2015-09-17

**Authors:** Andrew P. J. Olson, Bernard Trappey, Michael Wagner, Michael Newman, L. James Nixon, Daniel Schnobrich

**Affiliations:** Department of Medicine, University of Minnesota Medical School, 420 Delaware St, MMC 741, Minneapolis, MN 55455 USA; Department of Pediatrics, University of Minnesota Medical School, Minneapolis, MN USA; Department of Medicine, Weill Cornell Medical College, New York, NY USA; Minneapolis VA Medical Center, Minneapolis, MN USA

**Keywords:** Point-of-care ultrasound, Diagnosis, Splenomegaly, Sensitivity, Specificity, Physical examination

## Abstract

**Background:**

It is important to detect splenomegaly as it can have important diagnostic implications. Previous studies, however, have shown that the traditional physical examination is limited in its ability to rule in or rule out splenomegaly.

**Objective:**

To determine if performing point-of-care ultrasonography (POCUS) in addition to the traditional physical examination improves the sensitivity and specificity for diagnosing splenomegaly.

**Methods:**

This was a prospective trial of diagnostic accuracy. Physical and sonographic examinations for splenomegaly were performed by students, residents and attending physicians enrolled in an ultrasound training course. Participants received less than 1 h training for ultrasound diagnosis of splenomegaly. The findings were compared to radiographic interpretation of gold standard studies.

**Setting/patients:**

Hospitalized adult patients at an academic medical center without severe abdominal pain or recent surgery who had abdominal CT, MRI or ultrasound performed within previous 48 h.

**Results:**

Thirty-nine subjects were enrolled. Five patients had splenomegaly (12.5 %). The physical examination for splenomegaly had a sensitivity of 40 % (95 % CI 12–77 %) and specificity of 88 % (95 % CI 74–95 %) while physical examination plus POCUS had a sensitivity of 100 % (95 % CI 57–100 %) and specificity of 74 % (95 % CI 57–85 %). Physical examination alone for splenomegaly had an LR+ of 3.4 (95 % CI 0.83–14) and LR− of 0.68 (95 % CI 0.33–1.41); for physical exam plus POCUS the LR+ was 3.8 (2.16–6.62) and LR− was 0.

**Conclusions:**

Point-of-care ultrasonography significantly improves examiners’ sensitivity in diagnosing splenomegaly.

## Background

Splenomegaly is an important clinical finding that can have significant diagnostic implications. Its presence may be an important clue to malignancy, infections, or inflammatory conditions. Conversely, the absence of splenomegaly can also be an important finding and influence the diagnostic evaluation.

Historically, the reference standard for evaluating diagnostic accuracy of tests for splenomegaly has been splenic scintigraphy [1] or ultrasonography [2–4], but more recently computed tomography has also been used as a reference standard. The physical examination [3] for splenomegaly includes palpation, percussion by Nixon’s method [5], percussion by Castell’s method [6], and percussion of Traube’s space [7]. These commonly used methods have sensitivity ranging from 11 to 85 % and specificity from 32 to 99 %.

Point-of-care ultrasonography (POCUS) involves the acquisition and interpretation of a narrow set of simple, often dichotomous, findings by a provider at the patient’s bedside [8]. Emergency physicians were the first in the United States to show that POCUS markedly improved the detection of important findings such as intra-abdominal fluid [9]. Further studies have demonstrated that focused training for a broad number of ultrasound skills during residency was feasible [10–13]. More recently, POCUS has become increasingly common in fields such as internal medicine and family medicine. The purpose of our study was to evaluate the use of POCUS in addition to the traditional physical exam for detection of splenomegaly. We hypothesized that, with limited training, point-of-care ultrasonography would outperform the traditional physical examination.

## Methods

**Fig. 1:**
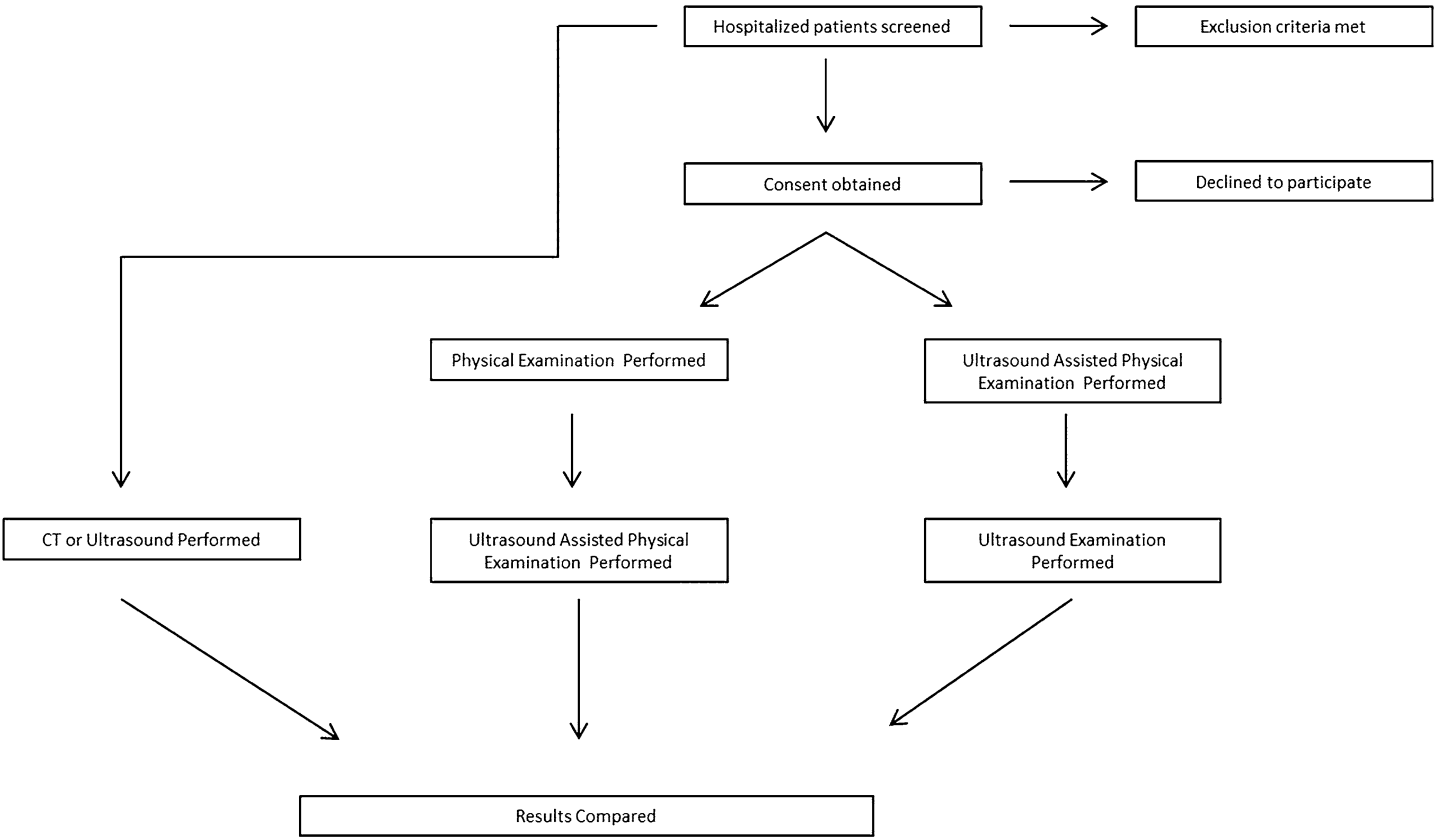
Study flow

Ultrasonographic and physical exam assessments for splenomegaly were performed on a convenience sample of patients admitted to the Hospital Medicine and Hematology/Oncology services at the University of Minnesota Medical Center between July 2013 and March 2015. One investigator screened the medical records of patients to identify those meeting the following criteria: (1) 18 years of age or older; (2) capable of giving informed consent; (3) lack of moderate-to-severe abdominal pain; (4) lack of abdominal surgeries in the last 30 days; (5) CT, MRI, or ultrasound of the abdomen had been performed in the course of usual clinical care within the last 48 h. Eligible patients were then approached and those who consented underwent ultrasound and physical exams as described below (Fig. 1).

Traditional physical examinations and physical examinations plus POCUS were performed by medical students, residents and attending physicians (examiners). The examiners included internal medicine residents and hospitalists participating in an ultrasound course at the University of Minnesota Medical School [13] and third and fourth year medical students in an advanced physical diagnosis elective course. The training for examiners included a 15-min didactic presentation on how to measure the spleen along with practice on a healthy model 2–3 times over the course of several days.

Examiners were randomly assigned to perform either the traditional examination alone or traditional exam in conjunction with POCUS. For each patient enrolled, one traditional physical examination and one physical examination plus POCUS were performed by different examiners in succession (neither examiner had knowledge of the others’ findings). Sonographic examinations were performed using a portable ultrasound machine (NanoMaxx, Sonosite, Washington, USA) phased array or abdominal probe.

Physical examinations of spleen size were performed according to the examiners’ usual clinical practice; no history-taking was allowed, and patients were not required to be fasting. Sonographic examinations of the spleen were performed by placing the transducer in the left upper quadrant and examining the entire spleen. The largest anteroposterior dimension of the spleen was identified and measured. Splenomegaly was defined as an anteroposterior dimension >13 cm [14]. At a later time another investigator recorded whether or not splenomegaly was noted in the formal radiology report and this information used as the reference standard for the study. All clinicians performing examinations were blinded to the patients’ medical history, medical record, and formal radiology results.

This study was approved by the University of Minnesota Institutional Review Board.

### Statistical analysis

Data were entered into a secure database (RedCap) [15]. Sensitivities, specificities, likelihood ratios, and confidence intervals were calculated for physical examination and for physical examination plus POCUS, with the formal radiological study serving as the reference standard. Fisher’s exact test was used to calculate statistical significance for diagnostic tests in 2 × 2 contingency tables (Graphpad, La Jolla, CA, USA).

## Results

A total of 39 subjects were enrolled in the study. Five patients had splenomegaly by the reference standard (12.5 %). Seven medical students, 16 residents and 1 attending physician performed examinations during the study.

For splenomegaly, physical examination had a sensitivity of 40 % (95 % CI 12–77 %) and specificity 88 % (95 % CI 74–95 %) (*p* = 0.161) while physical examination plus POCUS had a sensitivity of 100 % (95 % CI 57–100 %) and specificity 74 % (95 % CI 57–85 %) (*p* = 0.003). For physical examination alone for splenomegaly, the LR+ was 3.4 (95 % CI 0.83–14) and LR− 0.68 (95 % CI 0.33–1.41); for physical exam plus POCUS the LR+ was 3.8 (2.16–6.62) and LR− was 0 (Table 1).

**Table 1:** Traditional physical examination and ultrasound-assisted physical examination findings for splenomegaly

Condition	Reference standard	Test	*n* (patients)	Sensitivity (95 % CI) [TP/FN + TP]	Specificity (95 % CI) [TN/TN + FP]	*p* value*	LR+ (95 % CI)	LR− (95 % CI)
Splenomegaly	CT or US	Physical examination alone	39	0.40 (0.12–.77) [2/5]	0.88 (0.73–0.95) [30/34]	0.161	3.40 (0.827–13.98)	0.68 (0.33–1.41)
		Physical exam + POCUS		1 (0.57–1) [5/5]	0.74 (0.57–0.85) [25/34]	0.003	3.78 (2.16–6.61)	0

*TP* true positive, *FP* false positive, *FN* false negative, *TN* true negative, *LR+* positive likelihood ratio, *LR−* negative likelihood ratio, *CT* computed tomography, *US* ultrasound

* *p* value for Fisher’s exact test

## Conclusions

We show that, in keeping with previous studies, the ability of the traditional physical examination to detect splenomegaly is limited. Performing point-of-care ultrasound in addition to the traditional physical examination improves the sensitivity for splenomegaly. In patients for whom the presence of splenomegaly would significantly alter the differential diagnosis, clinicians should use POCUS in addition to their traditional exam, as a negative exam effectively rules out splenomegaly.

This study has a number of important limitations. First, the sample size is relatively small. Second, this was a single-center study at an academic medical center with a significant number of end-stage liver disease patients, and thus there may be some availability bias [16], though this should have affected the physical and ultrasound exams equally. In addition, no history was taken and history-taking influences the accuracy of the physical examination [17]. Finally, the radiologist interpreting the images may not have used the most accurate measurement methods in his or her interpretation of the radiologic study, leading to a suboptimal reference standard.

It should also be noted that while the sensitivity of POCUS was much higher than the physical exam alone, POCUS was not more specific in this study. It is important that clinicians performing this exam are aware of the limited specificity of this exam to guard against overconfidence in applying a positive result. It follows that POCUS should be used to detect splenomegaly only when this is a clinically important question for a given patient; its use as part of a standard examination for every patient or as a screening test would result in many false positives. Presence of splenomegaly on POCUS should be confirmed with standard radiologic tests if the finding has clinical importance.

In conclusion, the performance of point-of-care ultrasonography in addition to the traditional physical examination improves students’, residents’, and attending physicians’ ability to detect splenomegaly. Given the relative ease of training for POCUS, one may consider adding this as a standard part of the clinical evaluation for splenomegaly.

## Abbreviations

POCUS: point-of-care ultrasonography
LR (+): positive likelihood ratio
LR (−): negative likelihood ratio
